# MDEM: A Multi-Scale Damage Enhancement MambaOut for Pavement Damage Classification

**DOI:** 10.3390/s25175522

**Published:** 2025-09-04

**Authors:** Shizheng Zhang, Kunpeng Wang, Pu Li, Min Huang, Jianxiang Guo

**Affiliations:** Software Engineering College, Zhengzhou University of Light Industry, 136 Science Avenue, Zhengzhou 450000, China; zshizheng@zzuli.edu.cn (S.Z.); 332316040988@zzuli.edu.cn (K.W.); superlipu@163.com (P.L.); huangmin@zzuli.edu.cn (M.H.)

**Keywords:** pavement damage classification, MambaOut, multi-scale feature fusion, detail enhancement

## Abstract

Pavement damage classification is crucial for road maintenance and driving safety. However, restricted to the varying scales, irregular shapes, small area ratios, and frequent overlap with background noise, traditional methods struggle to achieve accurate recognition. To address these challenges, a novel pavement damage classification model is designed based on the MambaOut named Multi-scale Damage Enhancement MambaOut (MDEM). The model incorporates two key modules to improve damage classification performance. The Multi-scale Dynamic Feature Fusion Block (MDFF) adaptively integrates multi-scale information to enhance feature extraction, effectively distinguishing visually similar cracks at different scales. The Damage Detail Enhancement Block (DDE) emphasizes fine structural details while suppressing background interference, thereby improving the representation of small-scale damage regions. Experiments were conducted on multiple datasets, including CQU-BPMDD, CQU-BPDD, and Crack500-PDD. On the CQU-BPMDD dataset, MDEM outperformed the baseline model with improvements of 2.01% in accuracy, 2.64% in precision, 2.7% in F1-score, and 4.2% in AUC. The extensive experimental results demonstrate that MDEM significantly surpasses MambaOut and other comparable methods in pavement damage classification tasks. It effectively addresses challenges such as varying scales, irregular shapes, small damage areas, and background noise, enhancing inspection accuracy in real-world road maintenance.

## 1. Introduction

Transportation plays a pivotal role in national economic development, with a welldeveloped road network serving as one of its key infrastructural foundations. However, due to long-term high-frequency usage and untimely maintenance, roads are prone to various degrees of damage, which can reduce their service life and pose safety risks [1]. Therefore, the timely detection and assessment of pavement damage are of great significance for road maintenance and driving safety. Early pavement inspection primarily relied on manual on-site surveys and measurements. While this method offers a certain level of intuitiveness, it is labor-intensive and subject to variability due to inspectors’ professional expertise and subjective judgment, leading to potential uncertainty in results. With the technological advancement, sensors, radar, laser imaging systems, and 2D/3D camera equipment have been increasingly applied in pavement damage detection. These automated techniques not only significantly enhance detection efficiency but also improve the objectivity and accuracy of the results [2]. For instance, Wang et al. [3] proposed a pavement mean texture depth detection method based on 3D laser scanning sensors; Zheng et al. [4] designed a minimum spanning tree algorithm integrated with 3D laser scanning for pavement damage identification. Although these methods offer high accuracy, they demand high-performance hardware and are unsuitable for lightweight detection scenarios. Zhang et al. [5,6] employed vehicle-mounted sensors to detect pavement damage by analyzing the level of vehicle jolts when passing over damaged areas. However, this method is limited in scope as it only covers the wheel–path regions.

In recent years, with the rapid advancement of machine learning and deep learning technologies, an increasing number of researchers have adopted computer vision methods for pavement damage detection and classification. For example, Wang et al. [7] constructed a convolutional neural network (CNN) model based on 5000 images to detect cracks in asphalt pavements. Eisenbach et al. [8] designed a CNN architecture named ASINVOS for identifying five types of pavement distress. The model consists of seven convolutional layers, three pooling layers, and two fully connected layers. Kim et al. [9] developed a CNN model based on AlexNet, trained on images collected from the internet for crack detection, achieving both accuracy and recall rates above 90%. Additionally, the model could identify cracks from real-time video, with recall and accuracy reaching 81% and 88%, respectively. Zhang et al. [10] proposed a model based on CrackNet CNN, which enabled automatic pixel-level crack detection using 1800 3D images. Although deep convolutional neural networks (DCNNs) have shown promising results in damage segmentation tasks, CNNs are inherently limited in capturing long-range contextual information, making it difficult to effectively model multi-scale irregular damage features [11]. Moreover, due to the fixed size of convolutional kernels, the receptive field is constrained, allowing only local feature extraction, which may result in discontinuous crack predictions or false positives [12]. With the widespread adoption of Transformer architectures in computer vision, researchers have begun leveraging their global attention mechanisms to enhance the representational capacity of pavement damage images, achieving initial success. For instance, Tang et al. [13] proposed a weakly supervised visual Transformer model, PicT, built upon the Swin Transformer framework. They introduced a patch-label teacher module to dynamically generate pseudo-labels for learning discriminative features under weak supervision. Xu et al. [14] developed a Transformer model named DMTC for dense multi-scale feature learning, integrating a cross-attention mechanism to detect pothole patterns. Tong et al. [15] introduced the ES-Transformer model, which combines Dempster–Shafer theory with a Transformer backbone to improve segmentation accuracy and uncertainty calibration. Evaluated on the Crack500 dataset, the model achieved a mIoU of 59.85%, demonstrating strong overall performance. However, the computational complexity of Transformer architectures remains a challenge as their computational cost scales quadratically with input image size, limiting deployment efficiency in lightweight applications.

**Figure 1:**
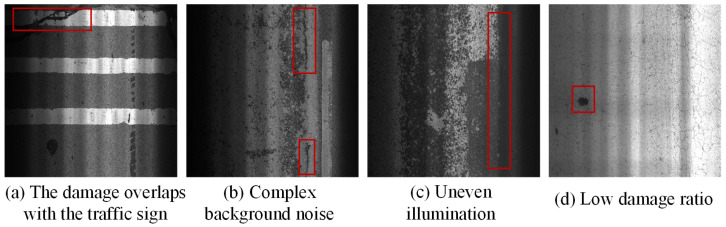
Four sample images from different pavement damage characteristics.

The aforementioned methods have proposed effective strategies for pavement damage detection and classification from various perspectives. However, restricted to the unique characteristics of pavement damage images, achieving high-precision and high-efficiency classification remains challenging. As shown in Figure 1, pavement damage in real-world scenes exhibits multi-scale and diverse patterns, ranging from fine cracks to large-area spalling. Irregular cracks have complex shapes, varying orientations, and discontinuous edges. They often overlap with traffic signs or shadows (a, b), increasing confusion between damage and background. Background regions frequently contain texture noise and granular contamination (b). Uneven illumination during image acquisition can further reduce the contrast between cracks and the background (c). In addition, low damage ratio is common (d), where damage occupies only a small area and is surrounded by large background regions, making it easily overlooked during feature learning. These factors collectively reduce the visual separability of different crack types, increase detection and classification difficulty, and limit the performance of existing methods in complex pavement scenarios.

**Figure 2:**
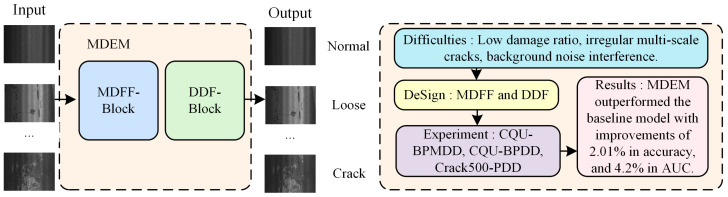
Schematic diagram of technical route.

This paper proposes a novel pavement damage classification model based on MambaOut [16], termed Multi-scale Damage Enhancement MambaOut (MDEM). MambaOut demonstrates excellent performance in image classification tasks due to its high modeling efficiency, low computational complexity, and hierarchical feature extraction mechanism. However, directly applying MambaOut to pavement damage classification presents two main limitations: First, it shows limitations in effectively modeling spatial structural details under complex backgrounds, which may hinder the extraction of discriminative features from fine-grained damaged regions. Second, it lacks an explicit mechanism for multi-scale feature fusion, potentially affecting its ability to recognize damage patterns with varying scales and visual similarities. To address these issues, we design MDEM. First, to improve its multi-scale modeling capability, a Multi-scale Dynamic Feature Fusion Block (MDFF) is designed, which combines multi-scale attention mechanisms with dynamic snake convolution to enable high-quality feature extraction for irregular damage across scales. Second, a Damage Detail Enhancement Block (DDE) is proposed, integrating spatial group enhancement and frequency-domain modeling to enhance the details of small-area damage regions and mitigate classification difficulties caused by background interference. The experimental results on the CQU-BPMDD dataset show that MDEM achieves improvements of 2.01% in accuracy, 2.64% in precision, 2.7% in F1-score, and 4.2% in AUC compared to the baseline MambaOut. The extensive experiments demonstrate that MDEM significantly outperforms MambaOut and other comparable methods in pavement damage classification tasks, offering an effective solution for damage recognition in complex scenarios. The technical route of this research is shown in Figure 2.

## 2. Related Work

### 2.1. Pavement Damage Classification

The pavement damage classification task aims to identify damaged regions in images and accurately classify them using visual methods. In recent years, to address the structural complexity and visual challenges of pavement damage images, researchers have proposed various solutions. For example, Zhang et al. [17] developed a model based on deep convolutional neural networks (ConvNet) capable of learning features directly from raw image patches. Liu et al. [18] proposed DeepCrack, a deep CNN model for pixel-wise crack segmentation, which integrates multi-scale features, deep supervision, and guided filtering to improve segmentation performance. Lin et al. [19] designed TransCrack based on the Transformer architecture, introducing a five-stage pure Transformer encoder and a contrastive learning attention mechanism (CLA). Through grid partitioning and positional embeddings, it captures long-range dependencies and fuses global and local attention at multiple scales, achieving high-resolution, pixel-level segmentation. Zhang et al. [20] proposed a classification model based on the Swin Transformer, enhancing representation capability through a fine-grained feature extraction module and attention re-embedding mechanism to improve classification accuracy. Blessing et al. [21] introduced Context-CrackNet, incorporating a region-focused enhancement module (RFEM) and a context-aware global module (CAGM) to strengthen the modeling of local details and global context. Sun et al. [22] designed a deep fusion selective scanning module (DFSS) within the DSWMamba framework to improve the receptive field and spatial localization ability. Tao et al. [23] proposed WSDD-CAM based on YOLOv5, which automatically generates pseudo-boundary boxes highly aligned with real boundaries via class activation maps (CAM), significantly reducing annotation cost and improving classification efficiency. Although CNN-based models have advantages such as fewer parameters and faster detection speed, they exhibit certain limitations in classification accuracy [24]. Transformer-based methods, while achieving higher accuracy, incur high computational costs and slower inference speed [25]. As an efficient alternative to Transformer architectures, Mamba [26] replaces the traditional self-attention mechanism with a bidirectional selective state space model (SSM), enabling efficient global modeling and incorporating positional embeddings to enhance spatial perception. However, for image classification tasks, long-sequence modeling is not essential. Thus, its simplified version, MambaOut [16], removes the SSM module and is more suitable for image classification scenarios. Based on this, we adopt MambaOut as the backbone and desgin Multi-scale Damage Enhancement MambaOut (MDEM) for pavement damage image classification. Considering that damages of different scales and shapes (e.g., alligator cracks, transverse cracks, and longitudinal cracks) are visually similar and prone to misclassification and that damaged regions often occupy small areas and overlap with traffic markings or shadows, further increasing classification difficulty, two key modules are designed: MDFF addresses the difficulty of distinguishing multi-scale damage by extracting fine-grained, high-quality features through multi-scale attention and dynamic snake convolution; DDE targets issues of low damage proportion and complex background interference by incorporating spatial group enhancement and frequency-domain information to improve the discriminability of damaged regions. The synergy between MDFF and DDE significantly improves the model’s classification accuracy in complex scenarios and demonstrates strong robustness and generalization capability.

### 2.2. Related Technologies

In recent years, deep learning has achieved remarkable progress in image restoration and reconstruction tasks. To address limitations in feature extraction capability, inadequate modeling of long- and short-range dependencies, and insufficient detail preservation, various efficient network architectures and feature enhancement mechanisms have been proposed. Qi et al. [27] introduced Dynamic Snake Convolution (DSConv), which incorporates topological and geometric priors to design controllable convolutional sampling paths for slender and tortuous structures (e.g., blood vessels and roads). Compared with conventional deformable convolution, DSConv enables more stable focus on fine structures and is further coupled with a multi-view feature fusion strategy that generates convolution kernels with diverse morphological templates, thereby enriching global structural representation from multiple scales and directions. Wang et al. [28] proposed the Multi-scale Attention Network (MAN) for super-resolution tasks involving both local texture and long-range dependency. Its Multi-scale Large Kernel Attention (MLKA) combines receptive fields of different sizes with a gating mechanism to dynamically fuse multi-scale attention maps, balancing global context modeling and local detail preservation. Zheng et al. [29] developed the Self-Modulation Feature Aggregation Network (SMFANet), which parallelly integrates an Efficient Approximation of Self-Attention (EASA) for non-local dependency modeling and a Local Detail Estimation (LDE) branch for high-frequency detail capture, achieving a favorable trade-off between accuracy and computational efficiency. Li et al. [30] proposed the Spatial Group-wise Enhance (SGE) module, which introduces spatial attention factors based on the similarity between global and local features within grouped convolutional channels, effectively suppressing noise and enhancing responses in taskrelevant semantic regions. Fourier frequency-domain modeling technique [31] exploits cross-correlation matrices to jointly capture multi-granularity relationships between global and local information, enabling adaptive channel weight allocation and improving the precision of feature representation.

Inspired by these advances, we address challenges in pavement damage classification. These include the high visual similarity of multi-scale cracks and different crack types, a low damage-to-background ratio, and complex background interference. We design the MDFF module to reduce classification confusion from scale variation and category similarity. We also propose the DDE module to increase the saliency of low-proportion damage regions and suppress background noise. Together, these modules strengthen damage feature representation and improve classification accuracy.

## 3. Methodology

**Figure 3:**
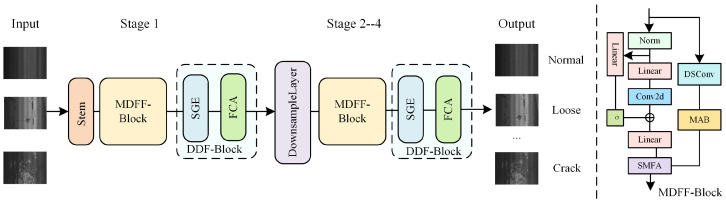
The overall architecture of MDEM.

In this section, a detailed explanation of the key components of MDEM is provided. Specially, Section 3.1 focuses on the MDFF, Section 3.2 elaborates on the DDE, Section 3.3 introduces the loss functions, and Section 3.4 presents the description of MDEM. The overall architecture of MDEM is illustrated in Figure 3.

### 3.1. Multi-Scale Dynamic Feature Fusion Block

**Figure 4:**
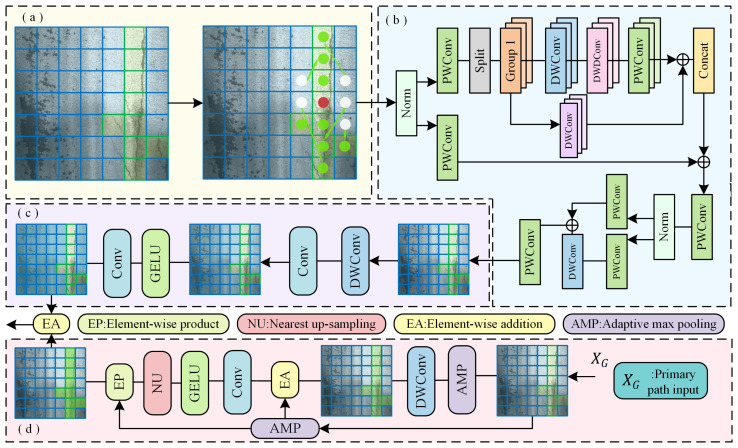
The architecture of MDFF. Part (**a**) is dynamic snake convolution; Part (**b**) is multi-scale attention mechanisms; Part (**c**) is used for nonlocal modeling; Part (**d**) is used for local modeling.

The structure of the Multi-scale Dynamic Feature Fusion Block (MDFF), as illustrated in Figure 4, is designed to address the limitations of the Gated CNN block in MambaOut with respect to feature extraction. When dealing with visually similar irregular crack damage, MambaOut primarily relies on hierarchical convolution for feature extraction, lacking deep modeling of crack shapes and structural details. This often results in insufficient feature representation and subsequent classification errors. To tackle these issues, we design the MDFF module by integrating dynamic snake convolution [27], multi-scale attention mechanisms [28], and both local and non-local feature interaction [29]. These components are specifically aimed at mitigating classification difficulties caused by high visual similarity in damage patterns.

When handling irregular crack damage, MambaOut can only perform coarse feature extraction, making it difficult to accurately capture features aligned with crack shapes. Unlike traditional fixed-kernel convolution, DSConv introduces topological geometric constraints to guide convolution kernels through continuous serpentine displacements. This enables the receptive field to remain concentrated on the target region, facilitating the effective extraction of elongated and curved cracks. Rather than relying on random offsets, this approach dynamically adjusts the sampling path based on the crack morphology, enabling the kernel to more precisely focus on local damage structures and significantly enhancing the representation of irregular cracks. This process is described by Equations (1)–(3), where $X_{DSC_{x}}$ and $X_{DSC_{y}}$ represent the new positions after offsetting along the *X* and *Y* axes, respectively. ($x_{i}$,$y_{i}$) and ($x_{j}$,$y_{j}$) denote the center positions of convolution, *c* is the step size, and △x and △y are the offsets along the *X* and *Y* axes. $X_{DSC}$ denotes the output feature value, while $X_{DSC}^{\prime }$ represents the original feature value used in interpolation. $B(X_{DSC},X_{DSC}^{\prime })$ is the weight contribution of $X_{DSC}^{\prime }$ to the interpolated target $X_{DSC}$, and $b(X_{DSC_{x}},X_{DSC_{x}}^{\prime })$ and $b(X_{DSC_{y}},X_{DSC_{y}}^{\prime })$ denote the weight contributions in the *X* and *Y* directions during interpolation.

(1) $$X_{DSC_{x}}=\left\{\begin{array}{c} x_{i}+c,\;y_{i}+\sum_{i}^{i+c}\Delta y\\ x_{i}-c,\;y_{i}+\sum_{i-c}^{i}\Delta y \end{array}\right.$$

(2) $$X_{DSC_{y}}=\left\{\begin{array}{c} x_{j}+\sum_{j}^{j+c}\Delta x,\;y_{i}+c\\ x_{j}+\sum_{j-c}^{j}\Delta x,\;y_{i}-c \end{array}\right.$$

(3) $$X_{DSC}=b\left(X_{DSC_{x}},X_{DSC_{x}}^{\prime }\right)\cdot b\left(X_{DSC_{y}},X_{DSC_{y}}^{\prime }\right)$$

Although MambaOut adopts a hierarchical architecture for progressive feature extraction, it lacks specific design for multi-scale crack modeling, resulting in limited capability when dealing with pavement damage of varying scales. By incorporating a multi-scale attention module, global dependencies can be captured using large convolution kernels, and parallel convolutional paths of different scales can be used to comprehensively perceive structural variations too. What is more, combined with a gating mechanism that focuses on local regions, the contribution of each scale’s attention map can be dynamically regulated to the final output, thereby resolving scale conflicts and enhancing sensitivity to high-frequency information such as texture edges and fine details at various scales. This process is described by Equations (4) and (5), where $X_{MAB}^{\prime }$ denotes the final output of the multi-scale processing module, LN(·) represents layer normalization, ⊗ denotes element-wise multiplication, $c_{i}$ represents the *i*-th point-wise 1×1 convolution layer, $f_{1}\left(\cdot \right)$ denotes the multi-scale large-kernel attention processing, and $f_{2}\left(\cdot \right)$ denotes the gating mechanism module. $\lambda_{i}$ is the learnable scaling factor used to balance the influence of the residual term on the final result.

(4) $$X_{MAB}=X_{DSC}+\lambda_{1}\cdot c_{3}\left(f_{1}\left(c_{1}\left(LN\left(X_{DSC}\right)\right)\right)⊗c_{2}\left(LN\left(X_{DSC}\right)\right)\right)$$

(5) $$X_{MAB}^{\prime }=X_{MAB}+\lambda_{2}\cdot c_{6}\left(f_{2}\left(c_{4}\left(LN\left(X_{MAB}\right)\right)\right),c_{5}\left(LN\left(X_{MAB}\right)\right)\right)$$

In addition, we adopt a feature interaction strategy that combines local and nonlocal modeling to enhance contextual representation and improve feature discrimination. First, high-frequency local modeling is applied to the features processed by dynamic snake convolution and the multi-scale attention mechanism, focusing on the texture edge information of cracks at different scales to enhance the expression of fine-grained details. Simultaneously, low-frequency non-local modeling is performed on the features processed by the Gated CNN block to extract global structural information beyond the damaged regions. Finally, a variance-based modulation mechanism is employed to adaptively guide the fusion of local and non-local features based on regional content variation, generating a more discriminative final output. This process is described by Equations (6) and (7), where $X_{MAB}^{\prime }$ is used for local modeling, $X_{G}$ is used for non-local modeling, and M(·) denotes the variance-based modulation mechanism for information fusion.

(6) $$\left\{X_{MAB}^{\prime },X_{G}\right\}=S\left(Conv\left({∥F_{in}∥}_{2}\right)\right)$$

(7) $$X_{MDFF}=M\left(X_{MAB}^{\prime },X_{G}\right)$$

Through the processing of the MDFF Block, the deficiency of MambaOut in feature extraction for visually similar, multi-scale irregular cracks can be effectively addressed. MDFF enhances the model’s attention to damaged regions and strengthens the representation of damage features, thereby providing strong support for achieving high-precision classification results.

### 3.2. Damage Detail Enhancement Block

**Figure 5:**
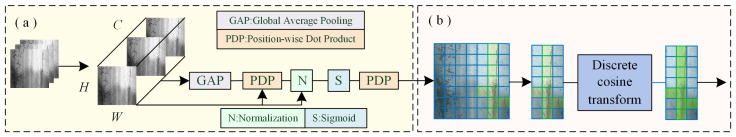
The architecture of DDE. Here, Part (**a**) is the spatial group enhancement mechanism; Part (**b**) is the Fourier frequency-domain modeling technique.

With respect to pavement damage classification tasks, the low proportion of damaged regions and complex background interference are two key challenges affecting performance. The former leads to only subtle differences between damaged and intact pavement images, increasing the risk of missed detections; the latter arises from frequent overlaps between damaged areas and elements such as shadows or traffic markings, which hinder accurate feature extraction and can result in misclassification. To address these issues, this paper designs the Damage Detail Enhancement Block (DDE), with its structure illustrated in Figure 5. This module integrates a spatial group enhancement mechanism [30] and Fourier frequency-domain modeling technique [31], aiming to improve the robustness and discriminability of feature representations, thereby effectively reducing background interference and enhancing classification accuracy.

Specifically, the spatial group enhancement mechanism first divides the channel features into multiple subgroups, allowing different submodules of the model to focus on distinct types of spatial features, thereby enhancing the expressiveness of local regions. Then, within each channel group, the global average of spatial positions is computed and used as the reference baseline for spatial attention generation. Through normalization and adaptive adjustment, attention factors are generated and applied to the original feature maps to weight and enhance key regions while suppressing irrelevant or interfering areas. Furthermore, DDE incorporates Fourier frequency-domain modeling to capture discriminative features in high- or mid-frequency components. This mechanism guides the network to focus on channel regions with significant spectral responses via frequency-domain analysis, further improving the model’s ability to recognize fine-grained damage. This process is described by Equations (8)–(13), where $X_{input}$ denotes the input feature to the spatial group enhancement mechanism, $x_{MDFF_{i}}$ represents local features within the *i* channel group, *g* denotes the global average feature within the current group, $c_{i}$ is the similarity coefficient, γ and β are attention control values for feature enhancement, σ(·) is the Sigmoid activation function, $X_{out}\left(x,y\right)$ represents the spatial-domain feature with positional information, ${\hat{X}}_{out}\left(u,v\right)$ denotes its frequency-domain representation, where (u,v) are frequency coordinates, $e^{-j2\pi (\cdot )}$ transforms spatial information into complex frequency responses, and $X_{DDE}$ corresponds to the features enriched with frequency-domain information.

(8) $$X_{input}=\left\{x_{MDFF_{1}},x_{MDFF_{2}},\cdot \cdot \cdot ,x_{MDFF_{m}}\right\}$$

(9) $$g=\frac{1}{m}\sum_{i=1}^{m}x_{MDFF_{i}}$$

(10) $$c_{i}=∥g∥\cdot ∥x_{MDFF_{i}}∥\cdot \cos\left(\theta_{i}\right)$$

(11) $$X_{out}=x_{MDFF_{i}}\cdot \sigma \left(\gamma \cdot c_{i}+\beta \right)$$

(12) $${\hat{X}}_{out}\left(u,v\right)=\sum_{x=0}^{H-1}\sum_{y=0}^{W-1}X_{out}\left(x,y\right)\cdot e^{-j2\pi (\frac{ux}{H}+\frac{uy}{W})}$$

(13) $$X_{DDE}=\left|{\hat{X}}_{out}\left(u,v\right)\right|$$

By introducing DDE Block, pavement damage features are enhanced, while background noise is suppressed, and frequency-domain information relevant to classification is incorporated. In this way, the problems of low damage ratios and background interference can be effectively handled.

### 3.3. Loss Function

In pavement damage image classification tasks, damaged regions typically occupy a small portion of the image, with complex backgrounds and high visual similarity among damage types. These factors make the model prone to overfitting during training, particularly when handling samples with blurred edges or ambiguous class boundaries, leading to unstable performance. To enhance the model’s robustness and generalization in such complex scenarios, we adopt the Soft Target Cross-Entropy loss function during training when using Mixup or CutMix data augmentation techniques. Unlike conventional cross-entropy loss based on one-hot encoding, the labels generated by Mixup and CutMix are probabilistic mixtures of multiple classes referred to as soft labels. Such labels better simulate real-world conditions involving ambiguous or mixed targets, guiding the model to learn more stably from uncertain or boundary-blurred samples. Formally, for a given input image with a soft target label $Y=\left\{y_{1},y_{2},\cdot \cdot \cdot ,y_{n}\right\}$ and predicted output $\hat{Y}=\left\{{\hat{y}}_{1},{\hat{y}}_{2},\cdot \cdot \cdot ,{\hat{y}}_{n}\right\}$, the loss is computed as Equation (14).

(14) $$l=-\sum_{i=1}^{K}y_{i}\cdot \log\left({\hat{y}}_{i}\right)$$

This loss design guides the model to construct smoother decision boundaries, effectively reducing the interference caused by label noise. It is particularly suitable for image samples with blurred damage edges, complex shapes, or extremely small damage ratios.

### 3.4. Multi-Scale Damage Enhancement MambaOut

The overall workflow of the Multi-scale Damage Enhancement MambaOut (MDEM) is as follows. When a pavement damage image is input into MDEM, it first passes through the stem layer for initial feature extraction. Then, the MDFF module is employed to fuse multi-scale damage information, followed by the DDE module for detail enhancement and frequency-domain modeling, improving the model’s sensitivity to small-scale and background-interfered damage features. This process constitutes Stage 1. Next, a downsampling operation is performed. Since downsampling reduces the spatial resolution of the feature maps and may lead to the loss of certain damage information, MDFF and DDE are re-applied to enrich damage features and enhance feature representation. These steps form Stages 2–4. After processing through all four stages, the fused high-level semantic features are passed into the classification head to generate the final classification prediction of the pavement damage image. This entire process is defined by Equations (15)–(17), where *X* denotes the input image; $X_{stage_{1}}$ is the output after Stage 1 processing; $S_{t}\left(\cdot \right)$ denotes the initial stem layer; d(·) denotes the downsampling layer; MDFF(·) and DDF(·) represent the MDFF and DDE modules; M(·) denotes the variance-based modulation mechanism for feature fusion; $f_{cls}\left(\cdot \right)$ represents the classification head; $Y_{i}$ denotes the final predicted classification result.

(15) $$X_{stage_{1}}=DDF\left(MDFF\left(S_{t}\left(X\right)\right)\right)$$

(16) $$X_{stage_{i}}=DDF\left(MDFF\left(d\left(X\right)\right)\right),2\leq i\leq 4$$

(17) $$Y_{i}=f_{cls}\left(X_{stage_{i}}\right)$$

In summary, we propose a novel pavement damage classification model named MDEM, which incorporates two key modules: MDFF and DDE. The MDFF module is designed to enhance the model’s ability to recognize visually similar, irregular, multi-scale cracks, while the DDE module guides the model to focus on low-proportion damage regions, enhancing damage details and reducing interference from redundant background information. Together, these components enable high-precision and accurate classification.

## 4. Experiments

### 4.1. Experimental Setting and Evaluation Metrics

All experiments in this work were performed on a 64-bit Windows 11 system. The hardware configuration comprised an Intel Core i5-12600KF processor (Intel, Santa Clara, CA, USA) and an NVIDIA RTX 4070 Ti SUPER GPU (Santa Clara, CA, USA) with 16 GB of memory. The model was developed using Python and built upon the PyTorch framework. CUDA version 11.8 was employed to accelerate training, thereby enhancing computational performance. In all experiments, the design of the fundamental parameters is shown in Table 1.

The experimental design of this study consists of two parts. The first part is a detection task, aiming to determine whether pavement damage exists in the input image. This task is evaluated using four commonly used metrics: AUC, F1-score, Precision, Recall, and FLOPs. The second part is a classification task, which focuses on identifying the specific type of damage. This task is also evaluated using four standard metrics: Accuracy, F1-score, Precision, Recall, and FLOPs. The specific calculation methods for each metric are detailed in Equations (18)–(22).

(18) $$Accuracy=\frac{TP+TN}{TP+TN+FP+FN}$$

(19) $$Precision=\frac{TP}{TP+FP}$$

(20) $$F1=2\times \frac{\frac{TP}{TP+FP}\times \frac{TP}{TP+FN}}{\frac{TP}{TP+FP}+\frac{TP}{TP+FN}}$$

(21) $$AUC=\sum_{i=1}^{n-1}\left(\frac{FPR_{i+1}-FPR_{i}}{2}\right)\times \left(FPR_{i+1}+FPR_{i}\right)$$

(22) $$Recall=\frac{TP}{TP+FN}$$

### 4.2. Dataset

**Figure 6:**
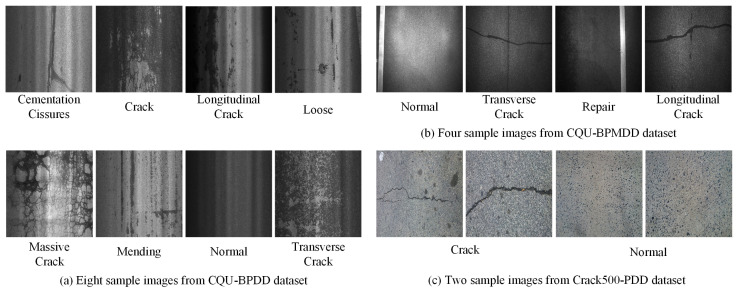
Example images of datasets.

To comprehensively and intuitively evaluate the performance of the MDEM model, three representative pavement image datasets are employed for experimental validation. First, CQU-BPMDD is the primary benchmark for comparison with mainstream image classification and pavement damage classification models. It contains 38,994 images (train/test split 7:3), including 9851 distress images covering longitudinal cracks, transverse cracks, repairs, and 29,143 normal images. Captured with exposure compensation, most images depict small-scale cracks, making detection more challenging and realistic. Second, CQU-BPDD serves as a supplementary dataset to assess generalization under different distributions. It consists of 60,056 asphalt pavement images collected across multiple South China locations under varied illumination and weather conditions. Pavement states are categorized into eight classes, with many cases featuring small damaged regions or lowlight conditions. The training set contains 10,137 images (5137 abnormal, 5000 normal), and the test set 49,919 images (11,589 abnormal, 38,330 normal). In addition, Crack500-PDD, a small-scale dataset, is used for ablation studies to evaluate performance in few-shot settings, further verifying robustness with limited data. The three datasets differ in scale, damage category coverage, and imaging conditions, enabling a multi-perspective assessment of MDEM’s effectiveness and adaptability. Detailed dataset statistics are provided in Table 2, with representative examples illustrated in Figure 6.

### 4.3. Ablation Analysis

To verify the individual contributions of the MDFF and DDE modules to the pavement damage classification task, as well as their combined effect, a systematic ablation study was conducted. In addition, we also conduct an experimental analysis of the loss function as well as the computational efficiency of the model. The experiments cover both detection and classification tasks, with results summarized in Table 3 and Table 4.

In the detection task, Table 3 shows that introducing either the MDFF or DDE module individually leads to significant improvements over the baseline model, indicating that both modules enhance feature representation from different perspectives. When the two modules are used in combination, the model achieves the highest AUC of 96.25%, representing a 4.2% improvement over the baseline, confirming the effectiveness of their synergy. Moreover, on the small-sample dataset, all evaluation metrics also show varying degrees of improvement, further demonstrating MDEM’s robustness and generalization capability under low-resource conditions.

In the classification task, as shown in Table 4, the integration of MDFF and DDE leads to comprehensive improvements across key metrics, including Accuracy, F1-score, Precision, and Recall. The overall performance significantly surpasses that of the baseline model, validating the effectiveness of the proposed architecture in multi-class fine-grained classification scenarios.

For the loss function, this study adopts the Soft Target Cross-Entropy loss (STCL). Compared with the standard cross-entropy, this approach is more suitable for pavement damage classification as it better exploits the similarity information between categories, mitigating classification instability caused by ambiguous class boundaries. The experimental results show that incorporating this loss function into MDEM yields noticeable performance improvements over the baseline. In terms of computational efficiency, although the introduction of the MDFF and DDE modules increases the FLOPs, substantial gains are achieved in key performance metrics such as accuracy. The trade-off between the accuracy improvement and the controllable increase in computational cost is both reasonable and worthwhile.

### 4.4. Detection Task Experimental Analysis

In the detection task, pavement damage classification is treated as a binary classification problem determining whether a damage region exists in the input image. To evaluate MDEM’s detection capability, it is compared against several representative pavement damage classification models, including PicT [13], DMTC [14], and DDACDN [32]. The experimental results are presented in Table 5. On the CQU-BPMDD dataset, MDEM outperforms both the baseline model and all comparative methods across all evaluation metrics, achieving a 4.2% improvement in AUC over the baseline. On the CQU-BPDD dataset, MDEM achieves likewise an AUC improvement of 1.85%, with leading performance in Precision, Recall, and F1-score. In addition, in terms of computational efficiency, MDEM is not inferior to other pavement damage classification models and even achieves certain improvements. These results collectively demonstrate that MDEM exhibits stable and superior detection performance under different data distributions, indicating strong generalization ability and adaptability to diverse pavement scenarios.

### 4.5. Classification Task Experimental Analysis

The classification task serves as a further refinement of the detection task, aiming to identify the specific type of damage present in an image. In this task, MDEM is first compared with a range of mainstream image classification models. The compared models include the following: AlexNet [33], VGG16 [34], MobileNetV2 [35], MobileNetV3 [36], FFVT [37], TransferG [38], Vision Mamba [39], Vision Transformer [40], and Swin Transformer [25]. The experimental results are presented in Table 6. On the CQU-BPMDD dataset, MDEM achieves notable improvements over the baseline model across multiple metrics: Accuracy increases by 2.01%, Precision by 2.64%, and F1-score by 2.7%. Moreover, MDEM outperforms all other mainstream image classification models across all metrics, further validating its effectiveness and advantage in complex pavement damage classification tasks. This indicates that the integration of MDFF and DDE modules effectively resolves the classification difficulties caused by multi-scale irregular damage, background interference, and low damage ratio.

**Figure 7:**
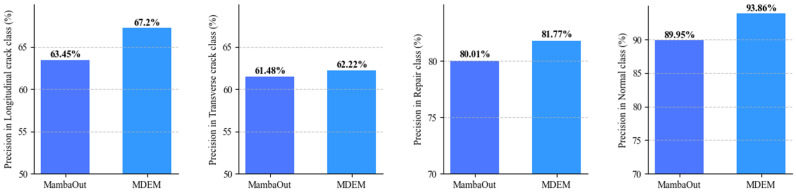
Comparison of different damage categories.

To further evaluate the model’s performance across different damage types, a per-class comparison was conducted between MDEM and the baseline model. As shown in Figure 7, MDEM outperforms the baseline across all damage categories, indicating stronger class discrimination ability and better adaptability.

In addition to comparisons with mainstream image classification models, MDEM was also evaluated against several task-specific pavement damage classification models. To comprehensively assess model performance and generalization capability, experiments were conducted under a dual-dataset setting, using both the CQU-BPMDD and CQU-BPDD datasets. This setup not only highlights MDEM’s performance advantage in standard scenarios but also verifies its robustness and generalizability under varying data distributions. The experimental results are presented in Table 7. The experimental results show that MDEM consistently outperforms other task-specific methods across key metrics including Accuracy, Precision, Recall, and F1-score on both datasets. Specifically, on the CQU-BPDD dataset, MDEM achieves a 1.95% improvement in Accuracy, a 1.24% increase in Precision, and a 1.5% gain in F1-score compared to the baseline model. In addition, in terms of computational efficiency, MDEM is not inferior to other pavement damage classification models and even achieves certain improvements. These findings further validate the proposed model’s universality and stability across different task contexts.

### 4.6. Visualization Analysis

**Figure 8:**
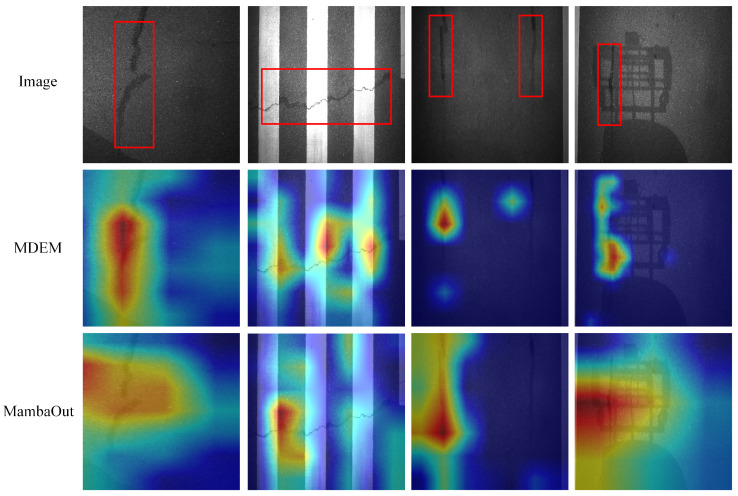
Visualized heat map of MambaOut and MDEM.

As shown in Figure 8, to further analyze the model’s focus during the classification process, we employ Grad-CAM [41] for heatmap-based visualization of the decisionmaking processes of both MDEM and MambaOut. On challenging samples, MDEM demonstrates a greater ability to accurately focus on the true damaged regions, exhibiting stronger localization and feature response capabilities. In contrast, MambaOut is more susceptible to background interference, with noticeable shifts in its attention regions. These results indicate that MDEM offers more stable discriminative region selection compared to MambaOut, which contributes to improved final classification accuracy.

**Figure 9:**
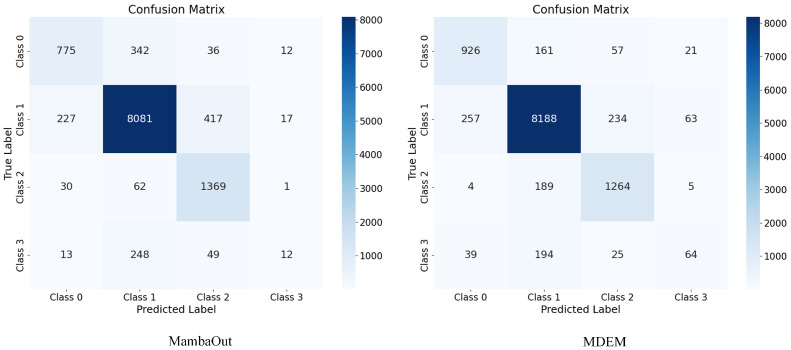
Confusion matrix of MambaOut and MDEM.

As shown in Figure 9, we compare the confusion matrices of MDEM and MambaOut. Overall, MDEM demonstrates higher classification accuracy under complex pavement conditions, with a noticeably lower misclassification rate, particularly in classes with low visual separability. When the number of samples is small, both methods experience a decline in accuracy; however, MDEM consistently achieves superior prediction performance. In most categories, MDEM produces a higher number of correct classifications than MambaOut, with misclassifications being more concentrated and less pronounced. In summary, compared with the baseline model, MDEM exhibits better performance in pavement damage classification tasks, offering stronger robustness and generalization capability.

## 5. Conclusions

This paper proposes a novel pavement damage classification model named MDEM. Built upon the MambaOut backbone, MDEM integrates two functional modules, MDFF and DDE, to address the challenges of recognizing multi-scale irregular cracks and detecting low-proportion damage regions in pavement images. The MDFF module enhances the model’s multi-scale feature modeling capability, while the DDE module improves detail representation. Although MDEM introduces additional computational overhead, the improvements in detection and classification performance are substantial, and the overhead remains controllable. The experimental results demonstrate that MDEM achieves outstanding performance across multiple datasets. Notably, it maintains stable and accurate classification even in scenarios with complex background interference and subtle damage regions. In summary, MDEM exhibits strong generalization, robustness, and practical applicability, providing effective technical support for pavement damage classification under complex traffic conditions. Future work will focus on reducing computational cost and model lightweighting, as well as extending the model to video-level applications for temporal detection and dynamic tracking of pavement damage.

## Figures and Tables

**Table 1:** Experimental parameter setting.

Parameter	Value
Learning Rate	0.00005
Weight decay	0.0005
Batch Size	32
Epoch	50

**Table 2:** The details of the datasets.

Dataset	Class	Number
CQU-BPMDD	Normal	29,143
	Longitudinal crack Transverse crack Repair	9851
CQU-BPDD	Normal	43,330
	Cementation issures Crack Longitudinal crack Loose Massive crack Mending Transverse crack	16,726
Crack500-PDD	Normal	268
	Crack	494

**Table 3:** Results of ablation experiments for the detection task.

Dataset	MDFF	DDE	STCL	AUC	Precision	F1-Score	Recall	FLOPs
CQU-BPMDD			√	92.4%	90.3%	90.5%	90.6%	2.31 G
	√		√	96.0%	92.95%	92.97%	93.01%	8.34 G
		√	√	95.89%	92.9%	92.82%	93.0%	2.31 G
	√	√		95.8%	92.6%	92.56%	92.8%	8.34 G
	√	√	√	96.25%	93.03%	93.03%	93.11%	8.34 G
Crack500-PDD			√	99.9%	99.4%	99.4%	99.4%	2.31 G
	√		√	99.9%	99.63%	99.58%	99.6%	8.34 G
		√	√	99.9%	99.47%	99.52%	99.56%	2.31 G
	√	√		99.9%	99.62%	99.6%	99.6%	8.34 G
	√	√	√	99.9%	99.73%	99.62%	99.71%	8.34 G

**Table 4:** Results of ablation experiments for the classification task.

Dataset	MDFF	DDE	STCL	Accuracy	Precision	F1-Score	Recall	FLOPs
CQU-BPMDD			√	87.56%	86.52%	86.60%	87.56%	2.31 G
	√		√	89.25%	88.68%	88.89%	89.25%	8.34 G
		√	√	89.26%	88.66%	88.86%	89.26%	2.31 G
	√	√		88.90%	88.43%	88.50%	88.90%	8.34 G
	√	√	√	89.32%	88.80%	88.93%	89.32%	8.34 G

**Table 5:** Experimental results of the detection task.

Dataset	Model	AUC	Precision	F1-Score	Recall	FLOPs
CQU-BPMDD	MambaOut [16]	92.4%	90.3%	90.5%	90.6%	2.31 G
	DMTC [14]	90.3%	88.4%	88.3%	88.4%	8.42 G
	DDACDN [32]	89.6%	87.9%	87.7%	87.9%	7.81 G
	PicT [13]	93.4%	90.6%	90.7%	90.8%	8.54 G
	MDEM	96.25%	93.03%	93.05%	93.11%	8.34 G
CQU-BPDD	MambaOut [16]	92.5%	90.4%	90.4%	90.5%	2.31 G
	DMTC [14]	90.8%	88.7%	88.1%	88.6%	8.42 G
	DDACDN [32]	90.2%	88.3%	87.9%	88.5%	7.81 G
	PicT [13]	93.6%	90.1%	90.3%	90.4%	8.54 G
	MDEM	94.21%	91.33%	91.37%	91.44%	8.34 G

**Table 6:** Experimental results are compared with the mainstream classification models.

Dataset	Model	Accuracy	Precision	F1-Score	Recall
CQU-BPMDD	MobileNetV3 [36]	83.15%	81.91%	82.23%	83.1%
	MobileNetV2 [35]	83.56%	81.88%	81.71%	83.6%
	Alexnet [33]	76.43%	75.16%	73.48%	76.34%
	VGG16 [34]	80.48%	80.63%	80.45%	80.48%
	Swin-T [25]	87.62%	88.56%	87.45%	87.7%
	ViT [40]	87.08%	86.96%	86.67%	87.1%
	ViM [39]	87.32%	88.21%	87.56%	87.26%
	MambaOut [16]	87.56%	86.52%	86.6%	87.56%
	FFVT [37]	82.56%	82.85%	81.62%	82.42%
	TransFG [38]	80.36%	80.87%	80.45%	80.36%
	MDEM	89.32%	88.8%	88.93%	89.32%

**Table 7:** Experimental results are compared with the same type of models.

Dataset	Model	Accuracy	Precision	F1-Score	Recall	FLOPs
CQU-BPMDD	MambaOut [16]	87.56%	86.52%	86.6%	87.56%	2.31 G
	DMTC [14]	84.32%	83.92%	82.68%	84.32%	8.42 G
	DDACDN [32]	81.62%	81.37%	80.93%	81.62%	7.81 G
	PicT [13]	87.32%	87.14%	87.21%	87.32%	8.54 G
	MDEM	89.32%	88.8%	88.93%	89.32%	8.34 G
CQU-BPDD	MambaOut [16]	87.37%	88.61%	87.63%	87.35%	2.31 G
	DMTC [14]	84.32%	83.92%	82.68%	84.3%	8.42 G
	DDACDN [32]	83.62%	83.73%	83.57%	83.61%	7.81 G
	PicT [13]	88.58%	88.14%	87.85%	88.55%	8.54 G
	MDEM	89.07%	89.71%	88.93%	89.07%	8.34 G

## Data Availability

The datasets analyzed during the current study are available from the corresponding author on reasonable request. Codes from the current study may be obtained from the corresponding authors upon reasonable request.

## Abbreviations

The following abbreviations are used in this manuscript:

MDEM	Multi-scale Damage Enhancement MambaOut
MDFF	Multi-scale Dynamic Feature Fusion Block
DDE	Damage Detail Enhancement Block
CNN	Convolutional Neural Network
DCNNs	Deep Convolutional Neural Networks
AlexNet	ImageNet Classification with Deep Convolutional Neural Networks
CLA	Contrastive Learning Attention mechanism
RFEM	Region-Focused Enhancement Module
CAGM	Context-Aware Global Module
DFSS	Deep Fusion Selective Scanning Module
WSDD-CAM	Weakly Supervised Damage Detection with Class Activation Mapping
CAM	Class Activation Mapping
SSM	Selective State Space Model
DSConv	Dynamic Snake Convolution
ViT	Vision Transformer
Swin-T	Swin Transformer
ViM	Vision Mamba
VGG16	Visual Geometry Group 16-layer network
FFVT	Feature Fusion Vision Transformer
TransFG	Transformer Architecture for Fine-Grained Recognition
DMTC	Dense Multi-scale Feature Learning Transformer
DDACDN	Deep Domain Adaptation for Pavement Crack Detection
PicT	Pavement image classification Transformer
STCL	Soft Target Cross-Entropy loss
